# Genome-Wide Association Studies Identify Heavy Metal ATPase3 as the Primary Determinant of Natural Variation in Leaf Cadmium in *Arabidopsis thaliana*

**DOI:** 10.1371/journal.pgen.1002923

**Published:** 2012-09-06

**Authors:** Dai-Yin Chao, Adriano Silva, Ivan Baxter, Yu S. Huang, Magnus Nordborg, John Danku, Brett Lahner, Elena Yakubova, David E. Salt

**Affiliations:** 1 Institute of Biological and Environmental Sciences, University of Aberdeen, Aberdeen, United Kingdom; 2 Department of Horticulture and Landscape Architecture, Purdue University, West Lafayette, Indiana, United States of America; 3 USDA–ARS Plant Genetics Research Unit, Donald Danforth Plant Sciences Center, St. Louis, Missouri, United States of America; 4 Center for Neurobehavioral Genetics, Semel Institute, University of California Los Angeles, Los Angeles, California, United States of America; 5 Gregor Mendel Institute of Molecular Plant Biology, Austrian Academy of Sciences, Vienna, Austria; Harvard University, United States of America

## Abstract

Understanding the mechanism of cadmium (Cd) accumulation in plants is important to help reduce its potential toxicity to both plants and humans through dietary and environmental exposure. Here, we report on a study to uncover the genetic basis underlying natural variation in Cd accumulation in a world-wide collection of 349 wild collected *Arabidopsis thaliana* accessions. We identified a 4-fold variation (0.5–2 µg Cd g^−1^ dry weight) in leaf Cd accumulation when these accessions were grown in a controlled common garden. By combining genome-wide association mapping, linkage mapping in an experimental F2 population, and transgenic complementation, we reveal that *HMA3* is the sole major locus responsible for the variation in leaf Cd accumulation we observe in this diverse population of *A. thaliana* accessions. Analysis of the predicted amino acid sequence of HMA3 from 149 *A. thaliana* accessions reveals the existence of 10 major natural protein haplotypes. Association of these haplotypes with leaf Cd accumulation and genetics complementation experiments indicate that 5 of these haplotypes are active and 5 are inactive, and that elevated leaf Cd accumulation is associated with the reduced function of *HMA3* caused by a nonsense mutation and polymorphisms that change two specific amino acids.

## Author Summary

Cadmium (Cd) is a potentially toxic metal pollutant that threatens food quality and human health in many regions of the world. Plants have evolved mechanisms for the acquisition of essential metals such as zinc and iron from the soil. Though often quite specific, such mechanisms can also lead to the accumulation of Cd by plants. Understanding natural variation in the processes that contribute to Cd accumulation in food crops could help minimize the human health risk posed. We have discovered that DNA sequence changes at a single gene, which encodes the Heavy Metal ATPase 3 (*HMA3*), drives the variation in Cd accumulation we observe in a world-wide sample of *Arabidopsis thaliana*. We identified 10 major HMA3 protein variants, of which five contribute to reduce Cd accumulation in leaves of *A. thaliana*.

## Introduction

Cadmium (Cd) is a significant pollutant and naturally occurring trace element that is potentially toxic to both plants and animals, including humans. The human body receives Cd from many sources, but mainly from food, drinking water and smoking [1]–[3]. An important step for Cd to enter the human food chain is its accumulation in plant tissues, especially the aerial parts that form the majority of the food sources consumed either directly by humans or through eating meat produced from animals raised on a plant-based diet [4]. High level accumulation of Cd in the harvestable, above-ground tissues of plants is also essential for successful phytoremediation of environments contaminated with potentially toxic concentrations of Cd [5]. Understanding the mechanism of Cd accumulation in plants is therefore an important step towards being able to control the health risk environmental Cd poses.

Accumulation of Cd in the aerial tissues of plants is determined by several factors, including the bioavailability of Cd in the soil, uptake from the soil solution by roots and radial transport within the root to the vascular system, translocation from the root, and storage in the above ground tissues. Plants take up Cd from the soil through the symplastic pathway, though apoplastic transport may also be important [6]. Translocation of Cd from the roots to the shoots requires loading of Cd into the xylem from the symplast in the stele. Xylem loading of Cd in plants requires the Heavy Metal ATPases AtHMA4 and/or AtHMA2 [7]–[13].

Unlike most animals, plant cells have large vacuoles that can be used for Cd detoxification through compartmentalization and storage. To date three types of transporters have been identified that are responsible for sequestering Cd into plant vacuoles. They are CAX-type antiporters, such as CAX2 and CAX4 [14], [15], the Heavy Metal ATPase 3 (HMA3) 16–19 and phytochelatin transporters ABCC1 and ABCC2 [20], [21]. Among these, the H^+^/Cd^2+^-antiporters and HMA3 transport the ionic form of Cd (Cd^2+^), whereas the phytochelatin transporters transport Cd chelated with phytochelatins [14]–[21]. Further, the various transport systems involved in the accumulation of leaf Cd play different roles. Heterologous expression of *AtCAX2* and *AtCAX4* in all tissues enhanced Cd accumulation in tobacco leaves [15], whereas selective expression only in roots decreased leaf Cd accumulation [14]. Similarly, *A. thaliana* plants over expressing *AtABCC1* and *AtABCC2* in all tissues accumulate higher leaf Cd than controls [20]. Such data suggest that enhancement of a root sink for Cd reduces foliar Cd accumulation where as an enhanced leaf sink can increase foliar Cd accumulation.

HMA3 shows high amino acid sequence similarity to both HMA2 and HMA4, but its function is distinct from either [10]. In contrast to the plasma membrane localization of both HMA2 and HMA4 [10], HMA3 is localized to the tonoplast [16]–[19]. Studies have established that HMA3 orthologs in many plant species function in sequestering heavy metals into the vacuole, but the metal specificity and their role in leaf Cd accumulation appear to vary. In rice, *HMA3* was identified as the responsible locus underlying a shoot Cd accumulation QTL [17]–[18]. Functional HMA3 was found to specifically restrict Cd accumulation in rice seeds and leaves [17]. *HMA3* is highly expressed in the Zn/Cd hyperaccumulators *Noccaea caerulescens* (previously named *Thlaspi caerulescens*) and *Arabidopsis halleri* [16], [22], suggesting it may play a positive role in Zn/Cd hyperaccumulation. Heterologous expression of *HMA3* from rice and *A. thaliana* in *Saccharomyces cerevisiae* (yeast) suggests that HMA3 can function to sequesters Cd into vacuoles [18], [23], whereas *HMA3* from *A. halleri* appears to function in Zn but not Cd detoxication [22]. Further, overexpression in *A. thaliana* of *HMA3* from *A. thaliana* enhanced Cd, Zn and Co tolerance and accumulation [19]. It is not clear if these differences in substrate specificity of HMA3 in the different species are a result of evolutionary divergence or the use of different experimental systems. The role of *HMA3* in regulating foliar Cd accumulation in *A. thaliana* also remains inconclusive. However, the overall evidence supports the conclusion that HMA3 functions at the tonoplast in vacuolar compartmentalization of multiple heavy metals including Cd, Zn, cobalt (Co) and lead (Pb) [16]–[19], [23].

Natural variation is a powerful resource for studying the molecular function of genes as well as understanding their ecological function [24]–[29]. Natural variation has been observed at *HMA3* in a limited number of species including rice, *N. caerulescens* and *A. thaliana* accessions [8], [16]–[19], and this variation has been established to impacts foliar Cd accumulation in rice and *N.* c*aerulescens*. However, to date population-wide variation in foliar Cd and the potential link with variation at the *HMA3* locus have not been investigated in any species. *Arabidopsis thaliana* is broadly distributed throughout the northern hemisphere growing in a diversity of climatic, edaphic and altitudinal habitats where it is likely to be exposed to a range of selective pressures [30]. The *A. thaliana* genome contains extensive diversity throughout its global range and at least part of this genetic diversity is associated with broad phenotypic variability [31], and also local adaptation [27]–[29]. This extensive natural variation in *A. thaliana* has also been utilized to uncover specific genes and QTLs involved in controlling natural variation in a variety of traits [24].

Traditionally, QTLs have been identified using experimental populations such as recombinant inbred lines (RILs) in which homozygous alternative alleles are segregating. These mapping populations have high power to detect QTLs because each allele is present in 50% of the recombinant lines. However, these populations are time consuming to develop and also suffer from low resolving power due to the limited number of recombination events that occur during their development. This leads to the identification of QTLs that span relatively large genomic regions, making identification of causal genes more difficult. Further, each mapping population is generated from a cross between two parental accessions potentially captures only two alternative alleles of any locus. This leads to very limited sampling of natural allelic diversity in a population and the low probability of detecting important minor alleles. An alternative approach to using experimental recombinant populations for QTL analysis is genome-wide association (GWA) mapping. This approach takes advantage of the large number of historic recombination events that have occurred within a population, and couples these events with linked DNA polymorphisms in order to associate phenotypic diversity with a relatively small region of the genome. However, unlike RIL populations where each allele is at a frequency of 0.5, in samples of natural populations rare alleles will occur at lower frequency making it difficult to detect their phenotypic effect. GWA mapping has been successfully used in *A. thaliana* [26], [32]–[37], rice [38]–[40]) and maize [41], [42] for the identification of QTLs and candidate genes for various ecological and agricultural traits. However, few if any of these studies have verified the candidate genes and polymorphisms identified using GWA mapping. Here, we report the use of GWA mapping for the identification of a major QTL for foliar Cd accumulation in *A. thaliana*. Further, we extend the GWA mapping with fine mapping in an experimental F2 population, genetic and transgenic complementation and with analysis of whole genome re-sequencing data for the identification of *HMA3* as the causal gene, and the identification of the specific protein coding haplotypes of *HMA3* that underlie natural variation in leaf Cd accumulation in the global *A. thaliana* population.

## Results

### Genome-wide association analysis of foliar cadmium accumulation in *A. thaliana*

In a previous GWA study using a population of 93 *A. thaliana* accessions we were unable to identify a major peak of linked SNPs associated with leaf Cd accumulation, though we did identify several SNPs with −log(*p*-value)>5 [31]. The absence of strong associations might be a result of the small population size used in this previous study combined with an underpowered experimental design (fewer control genoypes in each experimental block for inter block normalization). This is supported by the observation that in the Atwell et al. [31] study, which used a population of 93 accessions, only two SNPs linked to *HKT1* were observed to be significantly associated with leaf Na, whereas in an expanded population of 349 accessions Baxter et al. [26] observed 12 SNPs significantly associated with leaf Na and linked to *HKT1*. We therefore employed this enlarged mapping population of 349 accessions [26] for our current GWA study to identify reliable QTLs contributing to leaf Cd accumulation in the globally sampled *A. thaliana* population.

**Figure 1:**
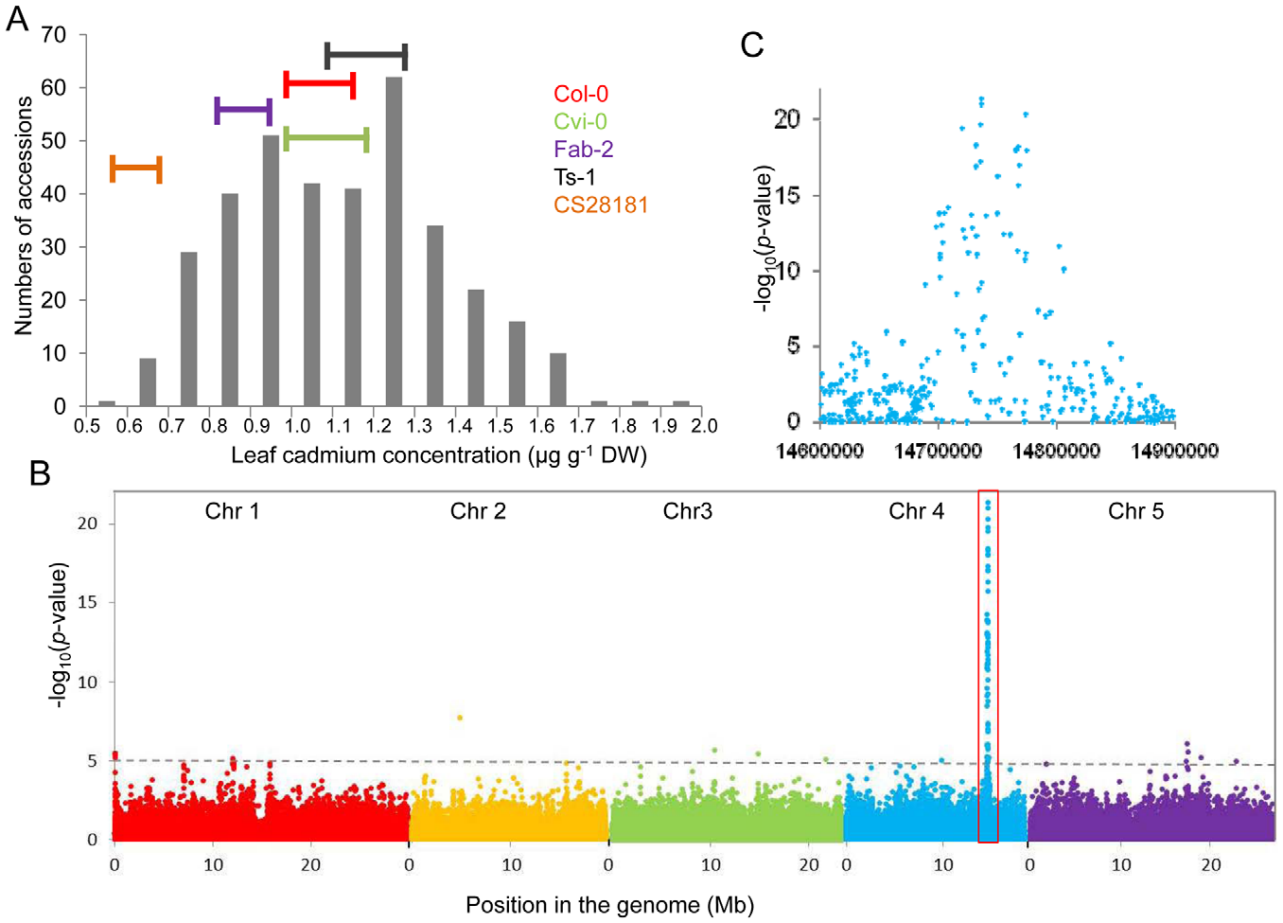
Genome-wide association analysis of leaf Cd accumulation in a worldwide collection of *A. thaliana* accession grown in a common garden. A. The frequency distribution of leaf Cd concentration in 349 *A. thaliana* accessions grown in a common garden. Horizontal bars represent the standard deviations of five accessions grown in all experimental blocks. B. Genome-wide association mapping of leaf Cd at 213,497 SNPs across 337 *A. thaliana* accessions using a mixed model analysis implemented in EMMA [33]. Horizontal dashed line indicates a genome-wide significance threshold of −log_10_ = 5. C. Detailed plot of the region shown in the red box in B.

Each accession was grown in a controlled common garden in potting mix soil with Cd supplied in the soil at a sub-toxic concentration of 90 µg kg^−1^. After 5-weeks of vegetative growth leaves were harvested individually from each plant and analyzed for Cd using inductively couple plasma mass spectrometry (ICP-MS) as described previously [43]. After normalization across experimental blocks using common genotypes and normalization of the ICP-MS data to an estimated leaf dry weight [26], we observed that leaf Cd concentrations varied across the 349 accessions from 0.5 to 2.0 µg g^−1^ dry weight (Figure 1A). From the 349 accessions 337 had previously been genotyped using the 256K SNP-tilling array Atsnptile 1, which contains a probe sets for 248,584 SNPs [26]. Using the genotype and leaf Cd concentrations for this subset of 337 accessions we performed a GWA analysis in which a population structure correction method implemented in EMMA was applied [31], [44]. In this genome-wide scan we observed a single region on chromosome 4 that contained multiple SNPs highly associated with leaf Cd concentrations (Figure 1B and 1C). In a 100 kb interval within this region we observed 54 SNPs significantly associated with leaf Cd (p-value<10^−5^), 39 of which were highly significantly associated with leaf Cd concentration (p-value<10^−10^). The most highly associated SNP was found at *Chr4:14736658* (−log (p-value) = 21.32), which explains 30% of the total variance in leaf Cd accumulation we observed. In contrast, no SNP contributing to more than 8% of the variance in leaf Cd was observed in any other region of the genome, suggesting the causal gene in linkage with SNP *Chr4:14736658* is the major genetic locus responsible for natural variation in leaf Cd accumulation in *A. thaliana*. At this peak SNP accessions with the cytosine (C) allele have leaf Cd on average 34.4% higher than accessions with thymine (T) allele. The minor allele (T) is represented in 42.4% of the population of 337 accessions. Within 40 kb either side of SNP *Chr4:14736658* (LD decay distance in this region) there are a total of 13 genes (Table 1), including *HMA2* and *HMA3*. Given that HMA2 and HMA3 have been shown to function as Cd and/or Zn transporters [10], [19], [23,], these two genes made good candidates for the causal gene underlying the observed Cd QTL centered on SNP *Chr4: 14736658*.

**Table 1:** Genes within 40 kb of the SNP most highly associated with leaf Cd.

Gene	start	stop	Direction	Annotation	Distance to SNP Chr4:*14736658*
AT4G30080	14703201	14706336	Reverse	ARF16, Auxin Response Factor 16	30322
AT4G30090	14708712	14711612	Reverse	emb1353, embryo defective 1353	25046
AT4G30097	14713518	14713661	Reverse	unknown protein	22997
AT4G30100	14714191	14720061	Forward	P-loop containing nucleoside triphosphate hydrolase	22467
**AT4G30110**	**14720241**	**14724584**	**Reverse**	**HMA2, Heavy Metal ATPase 2**	**12074**
**AT4G30120**	**14730401**	**14733510**	**Reverse**	**HMA3, Heavy Metal ATPase 3**	**3148**
AT4G30130	14734819	14737978	Forward	unknown protein	1839
AT4G30140	14738387	14740676	Reverse	CDEF1, Cuticle Destructing Factor 1	4018
AT4G30150	14742452	14749987	Forward	unknown protein	5794
AT4G30160	14753432	14760189	Forward	VLN4,Villin-Like actin-bindingprotein 4	16774
AT4G30170	14762841	14764627	Forward	Putative peroxidase	26183
AT4G30180	14768936	14769648	Forward	Putative transcription factor	32278
AT4G30190	14770499	14776056	Reverse	AHA2, Arabidopsis H(+)-ATPase 2	39398

### Geographic distribution of alleles at the SNP *Chr4:14736658*

To some extent, the geographic distribution of a genetic locus may reflect if there is selection for a particular allele in a certain environment. Using a genotyped worldwide collection of 1178 *A. thaliana* accessions in which the genotype at SNP *Chr4:14736658* is known, we plotted the geographical distribution of the two alleles at SNP *Chr4:14736658*. From this map we observe both alleles are widely distributed within Europe and central Asia and the USA (Figure S1). However, the enrichment of the two alleles varies by geographical region. For example, accessions with the T allele are enriched in the United Kingdom and western France, while accessions with the C allele predominantly occur in eastern Spain, eastern France, Germany, the Czech Republic and Sweden (Figure S1). The east-west structure in the geographical distribution of the alternate alleles at SNP *Chr4:14736658* in Europe may well be related to the known large-scale *A. thaliana* metapopulations that also have an east-west structure, related to range expansion from various southern glacial refugia [45].

### Linkage mapping of the Cd QTL in an experimental F2 population

**Figure 2:**
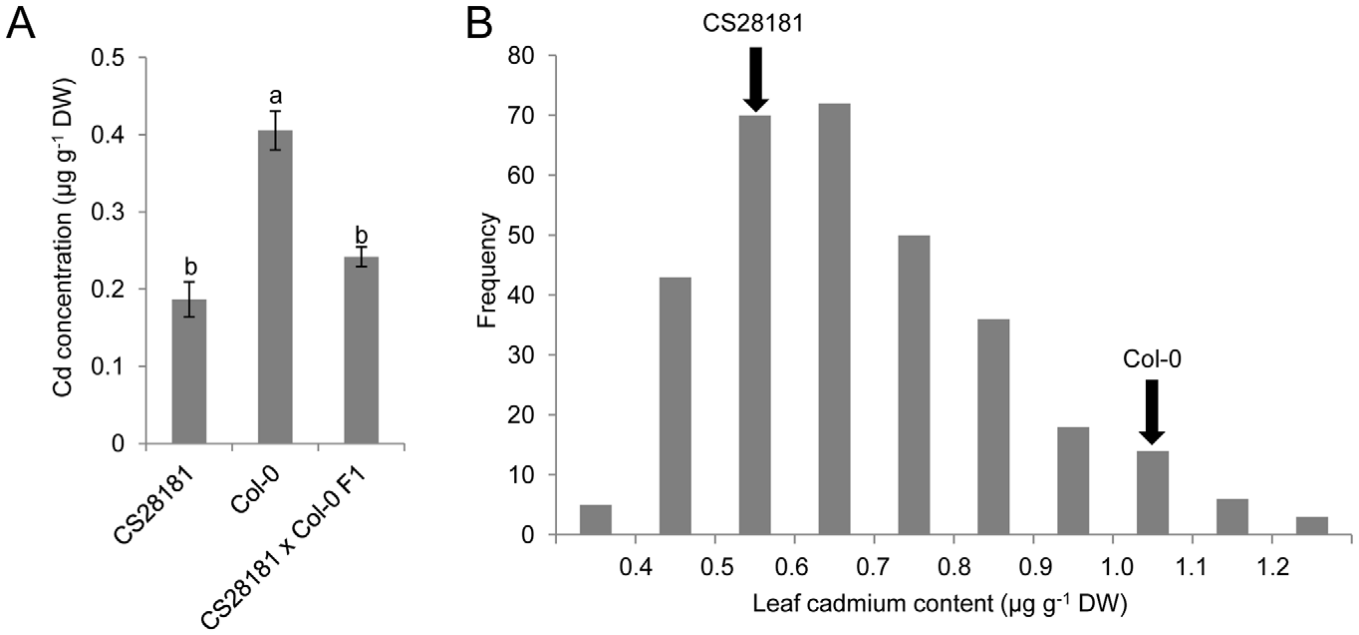
High leaf Cd in *A. thaliana* Col-0 is recessive to the low leaf Cd in the CS28181 accession. A. Leaf Cd concentration in *A. thaliana* accession CS28181, Col-0 and their F1 progeny. Data represent the mean leaf Cd concentration ± standard errors (n = 7–12 independent plants per genotype). B. The frequency distribution of leaf Cd concentration in F2 progeny of a cross between CS28181 and Col-0. Arrows indicate leaf Cd concentration of the parent accessions. Letters above each bar in (A) indicate statistically significant groups using a one-way ANOVA with groupings by Tukey's HSD using a 95% confidence interval.

**Figure 3:**
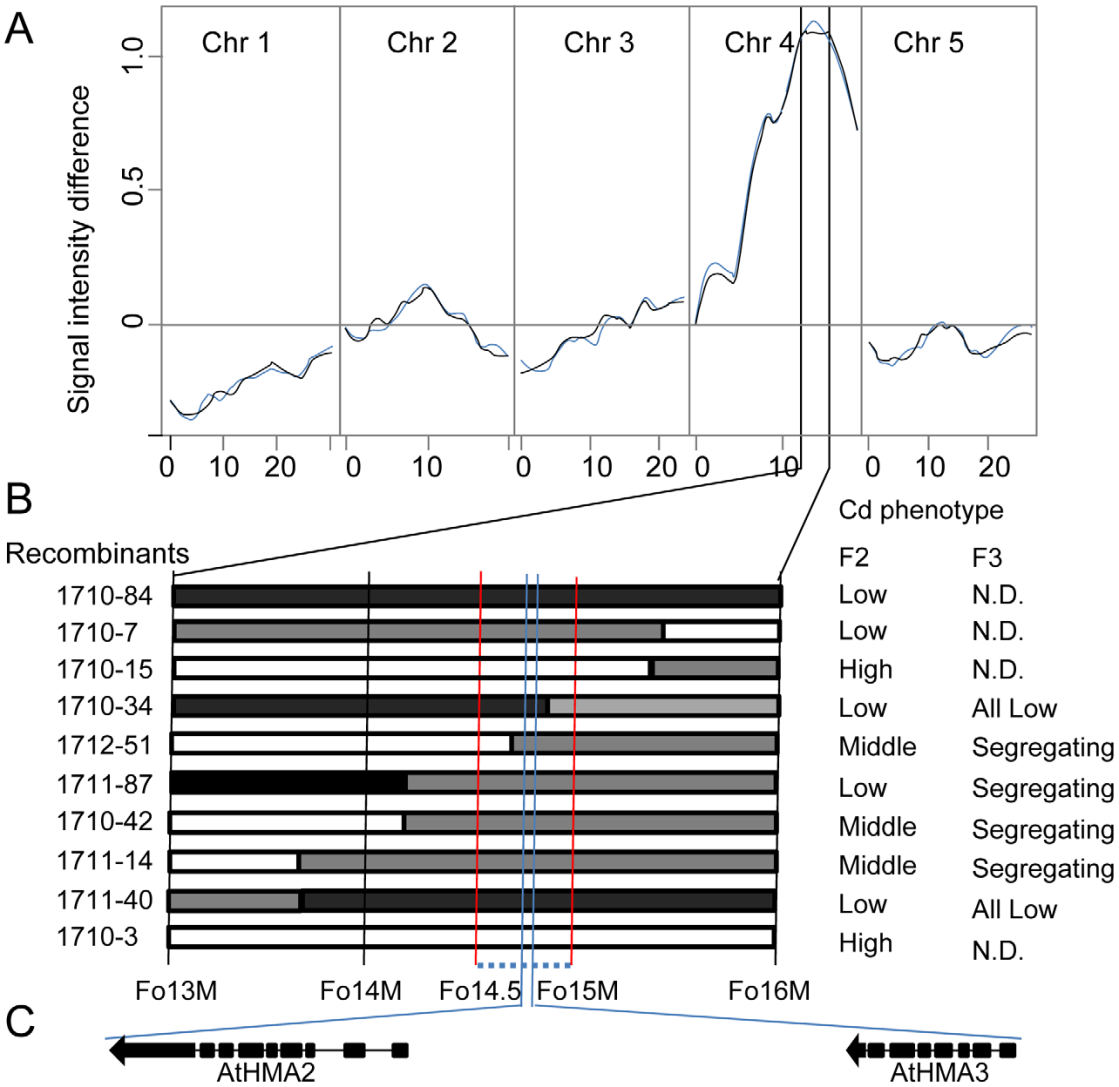
Genetic linkage mapping of the low leaf Cd locus in *A. thaliana* accession CS28181. A. DNA microarray-based bulk segregant analysis of the low leaf Cd phenotype of CS28181 using phenotyped F2 progeny from a CS28181×Col-0 cross genotyped using the 256K AtSNPtilling microarray. Lines represent allele frequency differences between high and low leaf Cd pools of F2 plants at SNPs known to be polymorphic between CS28181 and Col-0 (black line = sense strand probes, blue line = antisense strand probes). B. Fine mapping localizes the causal gene to a 500 kb interval between markers Fo14.5M and Fo15 indicated by the red vertical lines. Black bars represent the CS28181 genotype, grey bars represent heterozygous genotypes and white bars represent Col-0 genotype. Recombinants were selected from 317 CS28181×Col-0 F2 plants. Leaf Cd concentration was determined in the F2 and/or the F3 generation. C. Localization and gene structure of *HMA2* and *HMA3* in the mapping interval. Black bars indicate exons and black lines indicate introns.

To further genetically characterize the Cd QTL on chromosome 4 identified using GWA analysis we generated an experimental F2 population in which the alternate alleles of the diallelic SNP *Chr4: 14736658* were segregating. To achieve this we outcrossed the low leaf Cd *A. thaliana* accession CS28181, with a T at SNP *Chr4: 14736658*, to Col-0 which contains average leaf Cd and has a C at SNP *Chr4: 14736658*. The F1 generation of this cross had the same leaf Cd concentration as the CS28181 parent (Figure 2A), indicating that the CS28181 allele for leaf Cd accumulation is dominant over the Col-0 allele. eXtreme Array Mapping (XAM) was performed in which we combined bulk segregant analysis (BSA) with microarray genotyping [46], [47] using the CS28181×Col-0 F2 mapping population. A total of 314 F2 individuals in 4 experimental blocks were grown vegetatively in potting mix soil, leaves harvested after 5 weeks and analyzed by ICP-MS for Cd. Data was normalized across experimental blocks using the parental genotypes common within each block and normalization to estimated dry weight [26]. Consistent with the dominance of the CS28181 allele observed in the F1 generation the center of the distribution is shifted towards CS28181 leaf Cd accumulation (Figure 2B). 58 plants from the extreme high side of the Cd distribution (leaf Cd>0.85 µg g^−1^ dry weight) and 79 plants from the extreme low side of the Cd distribution (leaf Cd<0.55 µg g^−1^ dry weight) were pooled separately. Genomic DNA from each pool was isolated, labeled and hybridized to the Affymetrix SNP-tilling array Atsnptile 1. The allele frequency differences for all polymorphic SNPs were assessed according to hybridization signals as previously described [47]. Based on the allele frequency differences between the two pools, the causal locus of leaf Cd accumulation was mapped to a 3 Mb interval on chromosome 4 (from 13 Mb to 16 Mb) (Figure 3A), with the peak centered on the mapping interval identified in our GWA analysis (Figure 1B and 1C). The observation of a single strong XAM peak (Figure 3A) provides good supporting evidence for there being a single major QTL responsible for natural variation on leaf Cd accumulation.

PCR-based genotyping was used to further narrow down the mapping interval obtained using XAM. 314 F2 recombinants from the CS28181×Col-0 cross were individually genotyped at five CAPS markers spanning the 13–16 Mb interval on chromosome 4 and 20 recombinants between marker Fo13M and Fo16M were identified. According to the genotypes of these 20 recombinants and their leaf Cd accumulation in the F2 and/or F3 generations, we mapped the casual locus to a 500 kb region between marker Fo14.5M and marker Fo15M (Figure 3B), in which *HMA2* and *HMA3* are located (Figure 3C). Our linkage mapping in the CS28181×Col-0 mapping population confirmed the results we obtained from our GWA analysis, and further supported *HMA2* and/or *HMA3* as candidate genes driving the natural variation in leaf Cd accumulation we observed in our global *A. thaliana* population sample.

### DNA sequencing and trangenic complementation using *A. thaliana HMA2* and *HMA3*

As both association mapping and linkage mapping in *A. thaliana* indicate *HMA2* and *HMA3* are the best candidates for being responsible for natural variation of leaf Cd accumulation, we sequenced the genomic region covering the two genes in the accession CS28181, including the promoters, intergenic regions and 3′ termini. According to the assembled sequence, there are a total of 23 polymorphic sites between CS28181 and Col-0, of which 21 are SNPs and two are 1-bp deletion/insertions (Table 2). Of those polymorphic sites three are located in *HMA3* exons, three in *HMA2* exons, six in the *HMA3* promoter and two in the *HMA2* promoter. The polymorphisms in exons lead to differences of two amino-acid residues in HMA2 (Thr131Ala [CS28181 to Col-0 applied throughout] and Thr759Ala) and three amino-acid residues in HMA3 (Asn426Tyr, Ile448Arg and Leu543Stop). The premature stop codon in Col-0 *HMA3* is likely to eliminate the activity of the translated protein as it will be truncated after amino acid 542. The truncated product would lack the conserved ATP binding site and it is therefore likely to be non-functional [8]. It is also possible that the observed SNPs may contribute to differences in gene function between the CS28181 and Col-0 alleles.

**Table 2:** Polymorphisms in *A. thaliana HMA2* and *HMA3* between Col-0 and CS28181.

DNA nucleotide^a^		Position				Region affected	Amino acid residue	
Col-0	Cs28181	*HMA2G* b	*HMA2C* c	*HMA3G* b	*HMA3C* c		Col-0	CS28181
A	G	4235	2766	13160		Exon	C	C
C	T	3744	2275	12669		Exon	A	T
G	A	1506		10431		Intron		
C	T	868	391	9793		Exon	A	T
T	G	505		9430		Intron		
T	A	−391		8534		Promoter		
T	A	−392		8533		Promoter		
C	G	−3530		5395		Intergenic		
A	C	−3531		5394		Intergenic		
G	A	−4146		4779		Intergenic		
G	A	−4472		4453		Intergenic		
A	G	−5293		3632		Intergenic		
-	A	−6554		2371	1628	Exon	*	L
C	A	−6947		1978	1343	Exon	R	I
A	T	−7113		1812	1276	Exon	Y	N
G	A	−7467		1458		Intron		
A	G	−7727		1198		Intron		
C	A	−9226		−301		Promoter		
C	A	−9419		−494		Promoter		
C	G	−9524		−599		Promoter		
A	G	−9627		−702		Promoter		
T	C	−10155		−1230		Promoter		
A	-	−10155		−1230		Promoter		

a Nucleotide on the forward genomic strand.

b Indicates gDNA sequence.

c Indicates cDNA sequences.

* indicates stop codon. The position of nucleotides is relative to start codons of *HMA2* or *HMA3*.

**Figure 4:**
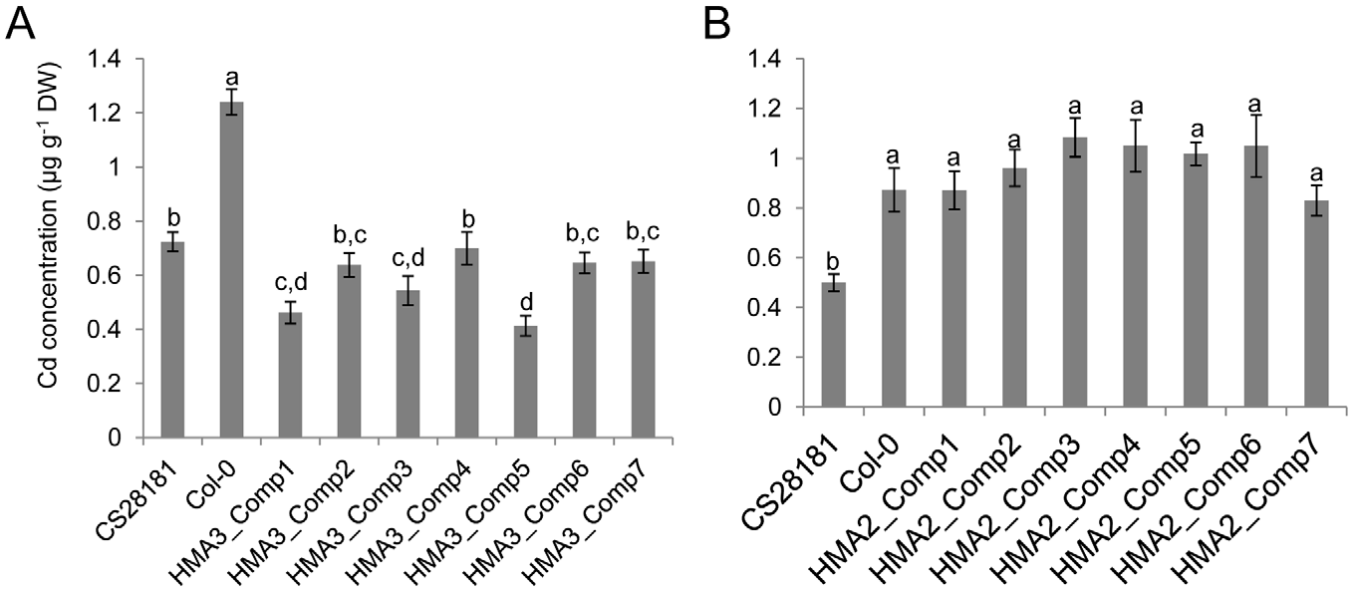
Transgenic complementation of the high leaf Cd phenotype of *A. thaliana* Col-0. The *A. thaliana* Col-0 accession was transformed with either CS28181 *HMA3* (A) or *HMA2* (B) and leaf Cd concentration determined. Transgenic complementation lines were made by introducing the CS28181 genomic DNA fragments of *HMA3* and *HMA2* (including promoter sequences) into the Col-0 accession. Data represents the mean leaf Cd concentration ± standard errors (n = 6–12 independent plants per genotype). Letters above bars indicate statistically different groups using a one-way ANOVA with groupings by Tukey's HSD using a 95% confidence interval.

Given that the sequence polymorphisms cannot exclude *HMA2* as a possible candidate gene, we used transgenic complementation to determine which gene underlies the observed leaf Cd QTL on chromosome 4. We constructed DNA vectors to introduce the CS28181 genomic DNA fragments of *HMA3* (*HMA3^CS28181^*) and *HMA2* (*HMA2^CS28181^*) separately into Col-0. These separate genomic DNA fragments included the 2 kb promoter region, the whole gene body and the 3′ terminal for both *HMA2^CS28181^* and *HMA3^CS28181^*. In the T2 generation, transgenic lines were grown vegetatively in potting mix soil, leaves harvested after 5-weeks and analyzed for Cd using ICP-MS. Because transgenic plants were segregating in the T2 generation, the reporter gene GUS was used as a marker for the transformation construct using histochemical staining in order to assess if an individual had the transgenic fragment. Individuals without the transgenic fragment were removed from further analysis. All seven independent Col-0 lines transformed with *HMA3^CS28181^* showed significantly reduced leaf Cd compared to Col-0 wild-type, with leaf Cd similar to, or even lower than CS28181 (Figure 4A). However, none of the Col-0 lines transformed with *HMA2^CS28181^* had reduced leaf Cd concentrations compared with Col-0 wild-type (Figure 4B). These results clearly indicate that it is *HMA3* and not *HMA2* that is the causal gene underlying the leaf Cd QTL we observe on chromosome 4.

### The role of the root and shoot in driving HMA3-controlled leaf Cd accumulation

**Figure 5:**
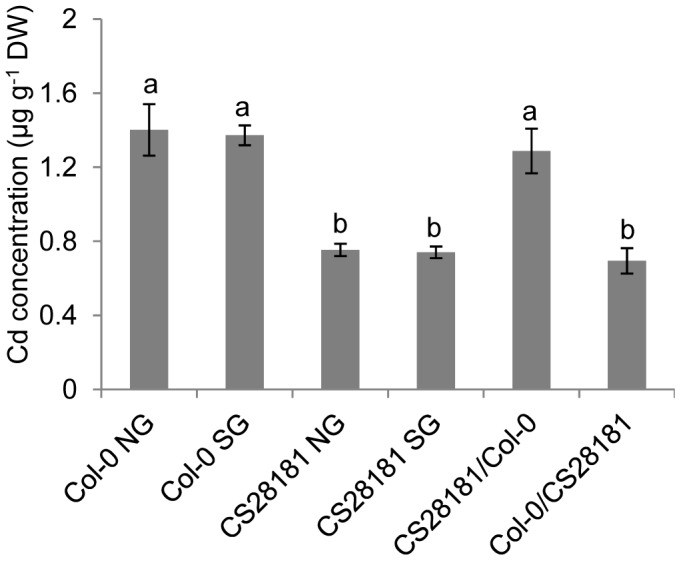
Reciprocal grafting determines that the low leaf Cd phenotype of CS28181 is driven by the root. Bars represent the leaf Cd concentration of reciprocally grafted *A. thaliana* CS28181 and Col-0 accessions. NG = Non-grafted plants; SG = Self grafted plants; CS28181/Col-0 = CS28181 shoot grafted onto a Col-0 root; Col-0/CS28181 = Col-0 shoot grafted onto a CS28181 root. Data represent means of leaf Cd concentration ± standard errors (n = 5–14 independent plants per grafting type). Letters above bars indicate statistically different groups using a one-way ANOVA with groupings by Tukey's HSD using a 95% confidence interval.

To determine which tissue (root or shoot) is responsible for controlling the *HMA3* dependent variation in leaf Cd between Col-0 and CS28181 we performed a reciprocal grafting experiment (Figure 5). Both self-grafted and non-grafted Col-0 had similar leaf Cd concentrations, as did the self-grafted and non-grafted CS28181. Further, both self-grafted and non-grafted CS28181 showed significantly lower leaf Cd than Col-0 (self-grafted or non-grafted) as expected. Grafted plants with a CS28181 root and a Col-0 shoot contained leaf Cd concentrations the same as CS28181 (self-grafted or non-grafted). Whereas, plants with a Col-0 root and a CS28181 shoot had leaf Cd concentrations the same as Col-0 (self-grafted or non-grafted). From this experiment we conclude that the variation in leaf Cd accumulation between Col-0 and CS28181, determined by *HMA3*, is driven by physiological processes in the root.

### Expression analysis of *HMA3* in *A. thaliana*

**Figure 6:**
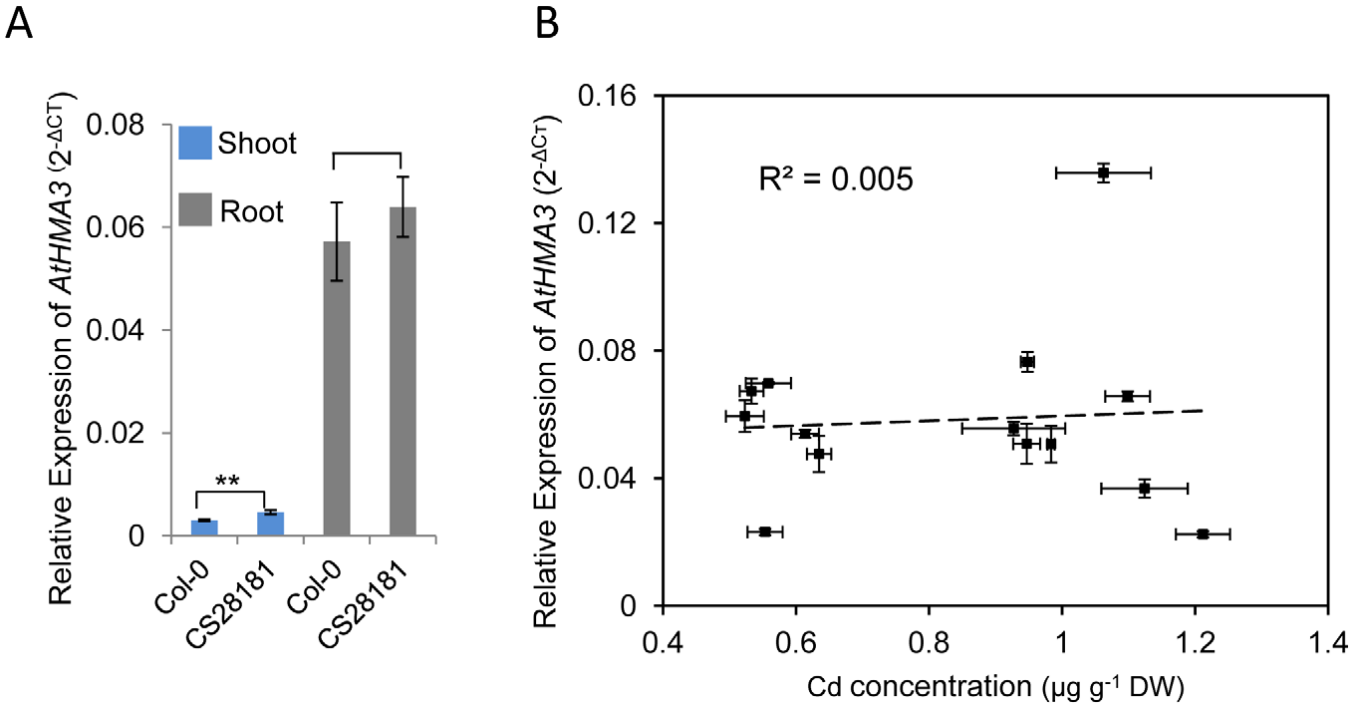
Quantification of expression of *HMA3* in various *A. thaliana* accessions by quantitative real-time RT–PCR. A. Expression of *HMA3* in shoot and root of *A. thaliana* accession Col-0 and CS28181. B. Correlation between root expression of *HMA3* and leaf Cd accumulation in 14 *A. thaliana* accessions. For the analysis *UBC* (*AT5G25760*) was used as an internal normalization standard across all samples. The expression of *HMA3* was calculated as 2^−ΔCT^ relative to *UBC*. Data represent means ± standard error (n = 4 independent biological replicates per accession and tissue).

Given that in many cases natural phenotypic variation is caused by cis-element polymorphisms driving changes in the level of gene expression [24], we used quantitative Reverse Transcription PCR (qRT-PCR) to quantify steady state levels of *HMA3* mRNA in Col-0 and CS28181. We observe that *HMA3* is primarily expressed in roots of Col-0, though we do observe expression in leaves to a lesser degree (Figure 6A). This is consistent with previous observations also using qRT-PCR [22]. Primary expression of *HMA3* in the root is also consistent with our observation that the root controls the *HMA3*-dependent variation in leaf Cd (Figure 5). However, we observe no significant difference between the steady state levels of *HMA3* mRNA in roots of Col-0 and CS28181 (Figure 6A). These results suggest that differences in the level of expression of *HMA3* between Col-0 and CS28181 cannot explain the differences in *HMA3*-dependent leaf Cd accumulation.

**Figure 7:**
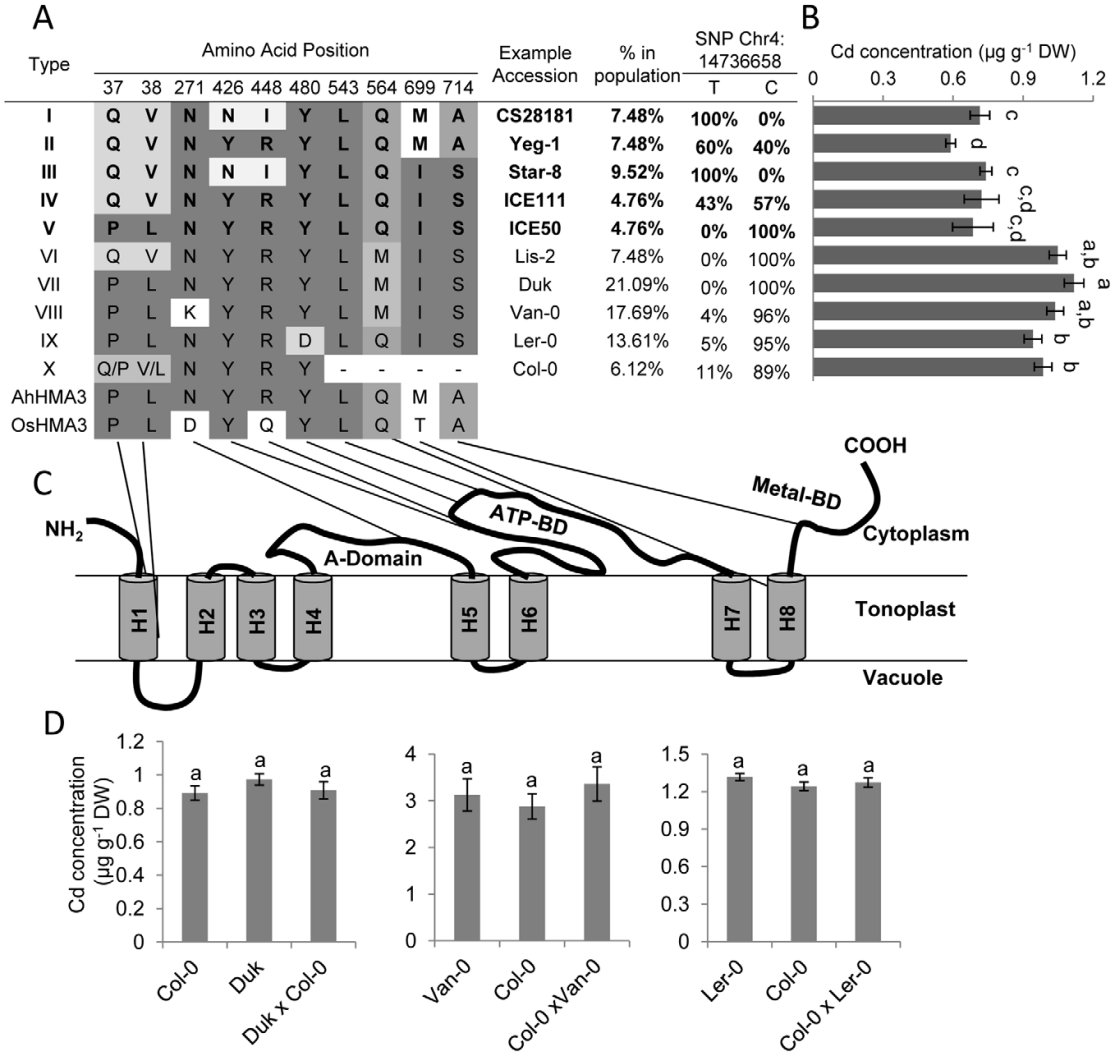
Natural *HMA3* protein coding haplotypes and their association with leaf Cd concentration across 149 *A. thaliana* accessions. A. Ten main *HMA3* protein coding haplotypes. B. Leaf Cd concentration of *A. thaliana* accessions grouped by *HMA3* protein coding haplotype. C. Predicted structural model of HMA3 with the position of the amino acid substitutions in (A) indicated in the model. H1–H8, transmembrane helixes. A-Domain = actuator domain; ATP-BD = ATP binding domain; Metal-BD = C-terminal metal binding domain. D. Leaf Cd concentration of F1 progenies of Duk×Col-0, Col-0×Van-0 and Col-0×Ler-0 and their parents in the same experiment. Data represents the means leaf Cd concentration ± standard errors (n = 5–19 in B, 12–20 in C). Letters right of the bars in (B) or above bars in (D) indicate statistically significant groups using one-way ANOVA with groupings by Tukey's HSD using a 95% confidence interval.

To further extend this analysis we used qRT-PCR to examine the steady state levels of *HMA3* mRNA in roots of 14 *A. thaliana* accessions grown on media solidified with agar (Figure 6B), representative of eight of the *HMA3* protein coding haplotypes we have identified (Figure 7) from a set of 149 re-sequenced accessions. We compared the root expression of *HMA3* to the leaf Cd accumulation in the same plants across all 14 accessions and observed that expression of *HMA3* varies among these 14 accessions but there is no correlation (R^2^ = 0.005) between *HMA3* mRNA levels and leaf Cd accumulation (Figure 6B). Further, we found a strong correlation between leaf Cd of the same accessions grown in potting mix soil and on agar solidified media (Figure S2). These results support our conclusion that root-driven *HMA3*-dependent variation in leaf Cd accumulation in *A. thaliana* is not due to variation in *HMA3* expression level.

### *HMA3* protein coding haplotypes across 149 *A. thaliana* accessions

Given that *HMA3* expression level polymorphisms do not appear to drive *HMA3*-dependent variation in leaf Cd in *A. thaliana* we investigated the possibility that this variation is due to differences in the function of the HMA3 protein. To test this hypothesis we examined the predicted protein coding haplotypes of HMA3 from a set of 149 genome re-sequenced *A. thaliana* accessions that we had previously phenotyped in potting mix soil grown plants for leaf Cd accumulation (Table S1). A total of 31 amino acid substitutions were found in the *HMA3* predicted amino acid sequence within this set of 149 genome re-sequenced accessions. Fourteen of those substitutions are only present in one accession (), which could represent sequencing errors. Seven of them are only found in 2–4 accessions, which are also considered as rare alleles. Removal of these 21 polymorphisms left 10 amino acid substitutions which were used to conservatively estimate the existence of 10 HMA3 protein coding haplotypes. (Figure 7A; Table S1). Given that the premature stop codon likely produces an inactive truncated HMA3 protein [8], [19] we put the two haplotypes with the 1-bp deletion causing the premature stop codon together and classify them as Type X (Figure 7A). We identified nine accessions in this class (Figure 7A). For each haplotype group we calculated the average leaf Cd concentration from leaf Cd data collected on all 149 accessions grown and analyzed individually (Figure 7B). A clear association between haplotype groups and leaf Cd concentration is observed, with accessions with haplotype I–V and haplotypes VI–X forming two distinctly separate low and high leaf Cd groups (Figure 7B).

Given that the group X haplotype is defined by a loss of function allele of *HMA3* [8], [19], we propose that elevated leaf Cd in this group is caused by loss of HMA3 activity. This is supported by the fact that the Col-0 allele of *HMA3* (which falls into haplotype group X) is a recessive allele compared to CS28181 (haplotype group I) (Figure 2). If elevated leaf Cd is associated with hypofunctional alleles of *HMA3*, such as the loss of function alleles in haplotype group X, then the haplotypes in groups VI, VII, VIII and IX are also likely to represent hypofunctional alleles of *HMA3*. Conversely, the CS28181 allele of *HMA3* is functional since it is dominant over the loss of function Col-0 allele (Figure 2), and therefore low leaf Cd is associated with hyperfunctional alleles of *HMA3* in protein coding haplotype groups I–V. Consistent with this, the Ws-2 allele of *HMA3*, which was previously established to be functional [19], falls into haplotype group III. To test our predicted functional classifications of the different *HMA3* protein coding haplotypes we examined leaf Cd accumulation in F1 plants from crosses between Col-0 and accessions with *HMA3* protein coding haplotypes VII, VIII and IX. None of these three haplotypes were able to complement the loss of function *HMA3* allele in Col-0 (Figure 7D), establishing that *HMA3* alleles in protein coding haplotype groups VII, VIII, IX are hypofunctional like the Col-0 allele.

Interestingly, the classification of the functional protein coding haplotype groups is consistent with our GWA study with the T allele at SNP *Chr4:14736658* being highly enriched in most of the functional haplotype groups, while the C allele is highly enriched in the hypofunctional groups (Figure 7A). However, this association is not perfect as might be expect for a linked yet non-causal polymorphism. We do though observe an absolute linkage between the *HMA3* protein coding haplotypes and function. The substitution of a glutamine at residue 564 (Q564) with a methionine (M564), or a tyrosine at residue 480 (Y480) with an aspartic acid residue (D480), are absolutely associated with loss of function of *HMA3*, reflecting the tight linkage between phenotype and genotype that would be expected for these putative casual polymorphisms.

A comparison of the functional *HMA3* orthologs in *Arabidopsis halleri* and rice [17], [22] with the functional *HMA3* protein coding haplotypes (I–V) in *A. thaliana* reveals that in *A. halleri* and rice the Q564 and M480 are also conserved, further supporting the conclusion that changes at these two residues in *A. thaliana* generate a non-functional HMA3 protein. The location of these two amino acid residues in the important ATP binding domain (Figure 7C) is also consistent with this inference.

### Role of HMA3 in regulating accumulation of other trace metals in *A. thaliana*

**Figure 8:**
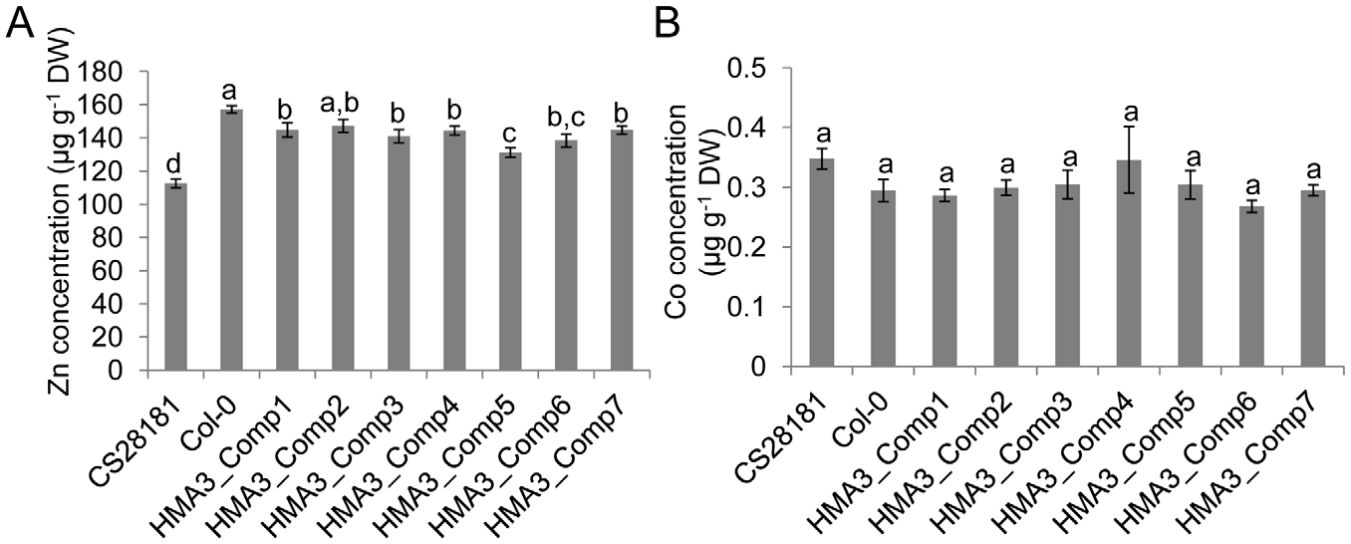
The effect of *HMA3* function on *A. thaliana* leaf Zn and Co. The *A. thaliana* Col-0 accession was transformed with CS28181 *HMA3* and leaf Zn (A) and Co (B) concentration determined. Transgenic lines were made by introducing the CS28181 genomic DNA fragment of *HMA3* (including promoter sequence) into the Col-0 accession. Data represents the mean leaf Zn and Co concentrations ± standard errors (n = 7–12 independent plants per genotype). Letters above bars indicate statistically significant groups using a one-way ANOVA with groupings by Tukey's HSD using a 95% confidence interval.

In rice, *HMA3* was found to specifically control leaf accumulation of Cd, but not Zn or other elements [17]. In *A. thaliana HMA3* was found to be involved in the transport of Cd and also possibly Zn, Co and Pb [19], [23]. To investigate a possible function for HMA3 in controlling variation in accumulation of these trace metals in *A. thaliana* we compared the foliar concentrations of Zn and Co in Col-0 (hypofunctional allele of *HMA3*) with CS28181 (hyperfunctional allele *HMA3*). A significant difference in leaf Zn was observed between Col-0 and CS28181 (Figure 8), with Col-0 having increased leaf Zn concentrations compared to CS28181. This elevated Zn was partially reduced by transformation of Col-0 with a genomic DNA fragment containing the CS28181 *HMA3* promoter, gene body and 3′ terminus (Figure 8). No significant differences in leaf Co were observed between CS28181, Col-0 or Col-0 transformed with the CS28181 *HMA3* genome clone (Figure 8). From these results we conclude that the hypofunctional allele of *HMA3* in Col-0 also affects the concentration of leaf Zn but has no effect on leaf Co.

## Discussion

Using GWA mapping on 349 *A. thaliana* accessions selected from a worldwide collections of 5810 accessions and genotyped at approximately 250,00 SNPs [26], [48] we successfully identified a single strong peak of SNPs associated with leaf Cd accumulation near to *HMA2* and *HMA3* (Figure 1B). The most highly associated SNP in this peak accounting for 30% of the total variance in leaf Cd after accounting for population structure. To confirm the GWA mapping result and identify the causal gene, we performed linkage mapping and transgenic complementation experiments and established that polymorphisms at *HMA3* are the major genetic determinant for the variation we observe in leaf Cd in this global *A. thaliana* population sample.

Expression level polymorphisms in *HMA3* do not appear to be responsible for the *HMA3*-dependent variation in leaf Cd we observe. This contrasts what we have previously found for natural variation in *A. thaliana* leaf Na and Mo levels which are driven by expression level polymorphisms in *HKT1* and *MOT1*, respectively [26], [49], [50]. In the reference accession Col-0 it had previously been observed that a 1-bp deletion in *HMA3* results in a premature stop codon, which was believed to cause a loss of function *HMA3* variant [8], [19]. However, the effect of this loss of function allele of *HMA3* on leaf Cd accumulation was not investigated, though a loss of function T-DNA insertion allele in Ws-2 was known to increase sensitivity to Cd [8], [19]. We compared the protein coding haplotypes of *HMA3* across 149 accessions and identified 10 major *HMA3* protein coding haplotypes. From the association of these haplotypes with leaf Cd concentrations in these accessions, and a comparison with the predicted amino acid sequence of *HMA3* from *A. halleri* and rice, we infer that five of the protein coding haplotypes of *HMA3* in *A. thaliana* are functional and the other five are non-functional. To confirm this hypothesis, we performed genetic complementation tests for 4 accessions representing 4 haplotype groups (I, VII, VIII and IX). Our results show that the known Col-0 loss of function protein coding haplotype cannot be complemented by *HMA3* alleles from haplotype group VII, VIII and IX (represented by Duk, Van-0 and Ler-0), which establishes that these three *HMA3* protein coding haplotypes also represent loss of function alleles. In contrast, the group I haplotype (represented by CS28181) is able to complement the loss of function allele in Col-0 confirming that this protein coding haplotype represents a functional allele of *HMA3*. From this we conclude that a major portion of the genetically determined natural variation in leaf Cd observed in our world-wide *A. thaliana* population sample is driven by variation in the level of function of the HMA3 protein. The sequence differences among active and inactive *A. thaliana* protein coding haplotypes of *HMA3* allowed us to further conclude that amino acid changes Gln564Met and Tyr480Asp are responsible for the impaired activity of the *HMA3* alleles in the protein coding haplotype group VI–IX. Furthermore, we could also confirm the previous observation that the premature stop codon is responsible for loss of function of protein coding haplotype group X. Interestingly, these amino acid changes occur in the ATP binding domain (Figure 7B) with Gln564Met and Tyr480Asp potentially affect ATP binding or ATP hydrolysis. Although further evidence is necessary to confirm the biochemical effects of these amino acids changes, our discoveries contribute to our understanding of the functional mechanism of HMA3 and other heavy metal ATPases.

It is interesting to note that the hypofunctional *HMA3* alleles we identify are more common than the hyperfunctional alleles in the genome re-sequenced population of 149 *A. thaliana* accessions we investigated. Ninety seven accessions contained hypofunctional *HMA3* protein coding haplotype, suggesting that the hypofunctional *HMA3* alleles are widely distributed in the *A. thaliana* population. This is supported by the high frequency and wide geographical distribution of the C genotype at SNP *Chr4: 14736658* linked to the hypofunctional *HMA3* allele. This raises the question of is the effect of the hypofunctional alleles of *HMA3* neutral or do they provide an adaptive benefit to the plant under certain environmental conditions? Recent genome-wide estimations of selection in *A. thaliana* did not reveal any evidence for selection at the *HMA3* locus [29]. However, it is possible that alleles could be adaptive in one environment but neutral in another [27]. Signals of selection of such locally adaptive alleles would be more difficult to identify in the world-wide *A. thaliana* sample used [29]. The adaptive function of these natural alleles of *HMA3* in Cd or Zn homeostasis, if there is any, remains unknown. We could speculate that the hypofunctional *HMA3* in *A. thaliana* might be neutral in soils with normal concentrations of Zn and beneficial in soils with low Zn where translocation of Zn to shoots needs to be maximized. Alternatively, the hyperfunational allele may be neutral in regions of low Cd and beneficial in areas of elevated Cd where enhanced vacuolar sequestration of Cd would potentially reduce the plants sensitivity to Cd. Further studies are required to eliminate the need for such speculation.

Genetics research indicates that loss of function of rice *HMA3* only affects accumulation of Cd, and not other heavy metals, and based on this observation it was concluded that HMA3 is a highly specific Cd transporter [17]. Our results presented here for *A. thaliana* are similar to rice in the sense that genetic alteration of *HMA3* function primarily effects Cd accumulation in leaves. However, unlike rice we observe in *A. thaliana* that *HMA3* also contributes to a lesser degree to leaf Zn accumulation. We would however caution against using such evidence to conclude that HMA3 in *A. thaliana* has higher specificity for Cd transport over other essential metals such as Zn. It is possible that the primary function of HMA3 in *A. thaliana* is in Zn transport, as has been proposed for HMA3 in *A. halleri* [22]. We observe that variation in *HMA3* function is not reflected in a large variation in leaf Zn accumulation in *A. thaliana* and propose this could be due to other genes involved in Zn homeostasis (e.g. *ZIPs*, *MTP1/3*, *HMA2/4*) responding to maintain normal tissue Zn concentrations. Because Cd accumulation in *A. thaliana* is unlikely to be tightly regulated in the same way that Zn is, variation in *HMA3* function is more clearly manifest in variation in leaf Cd accumulation. In a sense, variation in Cd accumulation is revealing hidden variation in Zn homeostasis mechanisms. However, further experiments are required to validate this model.

In previous studies in *A. thaliana*, *HMA3* has been shown to function in the detoxification of Cd [19], [23], but its role in limiting Cd translocation to the shoot was not investigated. We determine genetically that *HMA3* drives natural variation in leaf Cd concentration in *A. thaliana*, and grafting determined that *HMA3* functions in the root to determine leaf Cd concentration. Further, the known root expression pattern of *HMA3* is consistent with this observation. The expression pattern of *HMA3* in different plant species may be very important in determining its roles in regulating leaf Cd accumulation. Similar to *HMA3* in *A. thaliana*, rice *HMA3* is also predominantly expressed in root. Since *HMA3* functions in sequestering Cd into the vacuolar this expression pattern is consistent with *HMA3* acting to reduce leaf Cd accumulation in both *A. thaliana* and rice. In contrast, the Cd/Zn hyperaccumulators *N. caerulescens* and *A. halleri* express *HMA3* to extremely high levels in leaves where HMA3 is thought to enhance Cd sequestration into the vacuole, increasing its uptake [16], [22]. Consistent with this, constitutive over expression of a functional *HMA3* in *A. thaliana* increases leaf Cd accumulation two-fold [19].

In conclusion, our data supports a model of *HMA3* functioning in roots of *A. thaliana* to limit long-distance transport of Cd from root to shoot. We establish that the genetically determined natural variation in leaf Cd accumulation we observe in the *A. thaliana* global population is primarily controlled by variation of the function of *HMA3* driven by DNA polymorphisms in the protein coding region of the gene. Further, we propose there are two polymorphic amino acid residues and a nonsense mutation distributed among 10 protein coding haplotypes that drive this population-wide variation in *HMA3* function. These discoveries in *A. thaliana* improve our understanding of the mechanism of natural variation in Cd accumulation in plants. Further, they extend our knowledge of the function of *HMA3* which could contribute to the engineering or breeding of low Cd accumulating crop plants.

## Materials and Methods

### Plant materials and growth conditions

The 349 *A. thaliana* accessions for the GWA study were selected from 5810 worldwide accessions as described previously [26], [48]. 82 of the genome re-sequenced accessions used in this paper were obtained from the Arabidopsis Biological Resource Center. Most plants used for elemental analysis by ICP-MS were grown in a controlled environment [26], [43]. Briefly, seeds were sown on moist soil (Promix; Premier Horticulture) with non essential elements (As, Cd, Co, Li, Ni and Se) added at subtoxic concentrations in a 20-row tray. After stratification at 4°C for 3 days the tray was moved into a climate-controlled room for growth with a photoperiod of 10 h light (90 µmol·m^−2^·s^−1^)/14 h dark, humidity of 60% and temperature ranging from 19 to 22°C. Plants were bottom-watered twice a week with modified 0.25× Hoagland solution in which Fe was replaced by 10 µM Fe-HBED (*N,N′*-di(2-hydroxybenzyl)ethylenediamine- *N,N′*-diacetic acid monohydrochloride hydrate; Strem Chemicals, Inc.). Plants used for studying the relationship between expression of *HMA3* and leaf Cd concentration were grown in axenic conditions. Briefly, seeds were surface sterilized using 50% bleach and 0.05% SDS for 15 min, washed 8 times with sterilized deionized water and sown on ½ strength Murashige and Skoog (Sigma-Aldrich, St. Louis, USA) media solidified with agar containing 1% sucrose in Petri dishes. Plates were placed at 4°C for 3 days for seed stratification and then maintained at 16 h light (90–120 µmol·m^−2^·s^−1^)/8 h dark and 22°C. After 3-weeks growth, roots were harvested and used for RNA extraction and shoots were harvested for elemental analysis.

### Grafting of *Arabidopsis thaliana* plants

Seedlings were grafted as previously described [49]. Graft unions were examined before transfer to potting mix soil under the stereoscope to identify any adventitious root formation from graft unions or above. Healthy grafted plants were transferred to potting mix soil in a 20-row tray and grown in a controlled environment and after 4-weeks leaf samples were harvested as described above. After harvesting graft unions were examined again, and grafted plants with adventitious roots or without a clear graft union were removed from subsequent analysis.

### Elemental analysis

The determination of leaf elemental concentrations was performed as described previously [43]. One to two leaves (∼2–4 mg dry weight) were harvested from *A. thaliana* plants grown vegetatively for 5 weeks, leaves were rinsed with 18 MΩ water and placed into Pyrex digestion tubes. Samples were placed into an oven at 92°C to dry for 20 hours. After cooling, 7 reference samples from each planted block were weighed. Subsequently, all samples were digested with 0.7 ml concentrated nitric acid (OmniTrace; VWR Scientific Products) and diluted to 6.0 ml with 18 MΩ water. Gallium (Ga) was added in the acid prior to digestion to serve as an internal standard for assessing errors in dilution, variations in sample introduction and plasma stability in the ICP-MS instrument. Analytical blanks and standard reference material (NIST SRM 1547) were digested together with plant samples in the same manner. After samples and controls were prepared, elemental analysis was performed with an ICP-MS (Elan DRCe; PerkinElmer) for Li, B, Na, Mg, P, K, Ca, Mn, Fe, Co, Ni, Cu, Zn, As, Se, Mo and Cd. All samples were normalized to calculated weights, as determined with a heuristic algorithm using the best-measured elements, the weights of the seven weighed samples and the solution concentrations, detailed at www.ionomicshub.org. For GWA analysis data was normalized using common genotypes across experimental blocks as previously described [26], and this normalized data has been deposited on the iHUB (previously known as PiiMS [51]) for viewing and download through www.ionomicshub.org.

### Association mapping

The selection and genotyping of accessions for GWA analysis was described previously [26], [48]. Briefly, 5810 *A. thaliana* accessions were collected worldwide and genotyped at 149 genome-wide SNPs [26], [48]. These accessions were classified into 360 groups based on their genotypes at the 149 SNPs. One accession from each group was chosen to make a core set with 360 accessions. Among the core set of 360 accessions, 349 were phenotyped by ICP-MS for ionomic traits. Of this phenotyped subset 337 accessions were genotyped for at least 213,497 SNPs using the custom-designed SNP-tilling array Atsnptile 1 [26], [31], [48]. The GWA analysis was done using a linear mixed model to correct confounding by population structure [44] implemented in the program EMMA (Efficient Mixed-Model Association), which was described previously [31].

### Linkage mapping analysis

The SNP-Tilling array-based eXtreme Array Mapping (XAM) was done following the description of Becker et al. [47]. First, F2 progeny from an outcross of CS28181 and Col-0 were sorted by leaf Cd concentration. Approximately 25% of the total progeny at each end of the leaf Cd concentration distribution were pooled separately. From these pools approximately 300 ng genomic DNA was labeled separately using the BioPrime DNA labeling system (Invitrogen) and hybridized to the Affymetrix SNP-tilling array Atsnptile 1. The CEL files containing raw data of signal intensity for all probes were read and spatially corrected using R scripts from Borevitz et al. [52] with the R program and the Bioconductor Affymetrix package. The original CEL files used in this study have been submitted to the Gene Expression Omnibus (GEO) under accession GSE39679. Polymorphic SNPs between the two parents identified previously [52] were used for further analysis. There are 4 probes for each SNP, antisense and sense probes for two alleles. The allele frequency difference between the two pools for each SNP was then assessed based on the signal intensity difference of the 4 probes. The whole process can be carried out using R scripts that are available at http://ars.usda.gov/mwa/bsasnp [47].

PCR-based genotyping was used to further narrow down the mapping interval for the leaf Cd accumulation QTL. All 312 F2 plants that were phenotyped by ICP-MS were genotyped individually at 5 cleaved-amplified polymorphic sequence (CAPS) markers. The primers and restriction enzymes for the CAPS markers are listed in Table S2. Recombinants between marker Fo13M and Fo16M were selected for further analysis. The F2 recombinants with a clear low leaf Cd phenotype similar to CS28181 were directly used for determination of the candidate region. The F2 recombinants without a clear phenotype, or with a low Cd phenotype were selfed and 24 F3 progeny of each F2 individual further phenotyped for leaf Cd contetnt. According to the leaf Cd concentration of the F3's the genotype in the mapping interval was inferred and the region further narrowed.

### Sequencing of candidate genes and haplotype analysis

The candidate genomic region of CS28181 was sequenced through overlapping PCR. Firstly, 20 overlapping fragments were amplified using KOD hot start DNA polymerase (Novagen, EMD Chemicals, San Diego, CA USA) from the genomic region of CS28181 covering *HMA2* and *HMA3* and their promoters. The primers for the PCR reactions were designed using Overlapping Primersets (http://pcrsuite.cse.ucsc.edu/Overlapping_Primers.html) and are listed in Table S2. After purification, each fragment was sequenced using its amplification primers in two directions. The sequenced reads were assembled using SeqMan Lasergene software (DNASTAR; http://www.dnastar.com), with Col-0 sequence used as the reference. The *HMA3* haplotypes were analyzed using 149 genome re-sequenced *A. thaliana* accessions. Genomic sequence data of the 149 accessions was downloaded from the 1001 Genomes Data Center (http://1001genomes.org/data/MPI/MPICao2010/releases/2011_08_23/full_set/TAIR10, http://signal.salk.edu/atg1001/index.php,). The genomic sequences of the *HMA3* region were extracted using Text File Splitter 2.0.4 (http://www.softpedia.com/get/System/File-Management/Text-File-Splitter.shtml). The sequence data was introduced into Microsoft Excel and polymorphic nucleotides identified. The coding sequence (CDS) of each *HMA3* allele was predicted according to the reference cDNA of Col-0. Variations in protein amino acid sequence were identified according to the polymorphic nucleotides in the DNA sequence.

### Transgenic complementation

For construction of the expression vector of *A. thaliana HMA3* and *HMA2* two genomic DNA fragments for the two genes were PCR amplified from CS28181 using KOD hot start DNA polymerase and primers as listed in Table S2. The fragment for *HMA3* is ∼4.9 kb including 1.6 kb promoter region and 0.8 kb 3′ downstream sequence. The fragment for *AtHMA2* is ∼6.7 kb including 2.0 kb promoter region and 0.4 kb 3′ downstream sequence. The fragments were cloned into pCR-XL-TOPO vector (Invitrogen Life Technologies, http://www.invitrogen.com) for sequencing and subsequently recombined into binary vector pCAMBIA1301 by restriction enzymes of *Sal* I and *Bam*H I. The expression vectors with the two genes were transformed into *Agrobacterium tumeraciens* strain GV3101 and were introduced into Col-0 using the floral dip method [53]. Transgenic lines were screened on ½ strength Murashige and Skoog (Sigma-Aldrich, St. Louis, USA) medium solidified with agar containing 50 µg/ml Hygromycin and 1% sucrose.

### Quantitative real-time PCR

Total RNA was extracted from 3-week old plants grown on ½ strength Murashige and Skoog (Sigma-Aldrich, St. Louis, USA) medium solidified with agar containing 1% sucrose using TRIzol Plus RNA Purification kit (Invitrogen Life Technologies, http://www.invitrogen.com). Two microgram of total RNA was used to synthesize first strand cDNA with SuperScript VILO cDNA Synthesis Kit (Invitrogen Life Technologies, http://www.invitrogen.com). Quantitative real-time PCR was performed using SYBR Green PCR Master Mix (Applied Biosystems, USA) with the fist strand cDNA as a template on a Real-Time PCR System (ABI StepOnePlus, Applied Biosystems lco., USA). Primers for qRT-PCR were designed using Primer Express Software Version 3.0 (Applied Biosystems, USA). One primer of a pair was designed to cover an exon-exon junction. The primer sequences are shown in Table S2. Expression data analysis was performed as described previously [54].

## Supporting Information

Figure S1 Geographic distribution of accessions and their alleles at the SNP *Chr4:14736658*. Map showing the geographical position of the collection site of 1178 accessions of *A. thaliana*. The genotype at *Chr4:14736658* of each accession is represented by the type of symbol (red *Chr4:6392276* = C, yellow *Chr4:6392276* = T). Pie charts on the map represent the proportion of the local population containing the T allele (black sector) and C allele (white sector).Total 19 local populations (West USA, Middle-east USA, East USA, North UK, Middle UK, South UK, West France, East France, Portugal, Spain, Netherland, Middle Germany, North Germany, South Sweden, Middle Sweden, North Sweden, Czech Republic, The alps and South Italy) are plotted.
(TIF)

Figure S2 Comparison of the leaf Cd concentration in 14 *A. thaliana* accessions grown on different growth medium. Data represent the mean leaf Cd concentration ± standard errors (n = 4 for plants grown on solidified ½ MS media and 6–12 for plants grown on potting mix soil).
(TIF)

Table S1 Protein coding sequence variation of *HMA3* and leaf Cd concentration in 149 *A. thaliana* accessions.
(XLSX)

Table S2 Primers used in this study.
(XLSX)

## References

[1] Ursinyova M HV (2000) Cadmium in the environment of Central Europe. In: Markert Bernd A FK, editor. Trace Elements: their distribution and effects in the environment. 1 ed. Kindligton: Elsevier Science Ltd. pp. 87–108.

[2] Nawrot T, Plusquin M, Hogervorst J, Roels HA, Celis H, et al (2006) Environmental exposure to cadmium and risk of cancer: a prospective population-based study. Lancet Oncol 7: 119–126. 16455475 10.1016/S1470-2045(06)70545-9

[3] Verbruggen N, Hermans C, Schat H (2009) Mechanisms to cope with arsenic or cadmium excess in plants. Curr Opin Plant Biol 12: 364–372. 19501016 10.1016/j.pbi.2009.05.001

[4] Peralta-Videa JR, Lopez ML, Narayan M, Saupe G, Gardea-Torresdey J (2009) The biochemistry of environmental heavy metal uptake by plants: implications for the food chain. Int J Biochem Cell Biol 41: 1665–1677. 19433308 10.1016/j.biocel.2009.03.005

[5] LeDuc DL, Terry N (2005) Phytoremediation of toxic trace elements in soil and water. J Ind Microbiol Biotechnol 32: 514–520. 15883830 10.1007/s10295-005-0227-0

[6] Lux A, Martinka M, Vaculik M, White PJ (2011) Root responses to cadmium in the rhizosphere: a review. J Exp Bot 62: 21–37. 20855455 10.1093/jxb/erq281

[7] Wong CK, Cobbett CS (2009) HMA P-type ATPases are the major mechanism for root-to-shoot Cd translocation in Arabidopsis thaliana. New Phytol 181: 71–78. 19076718 10.1111/j.1469-8137.2008.02638.x

[8] Hussain D, Haydon MJ, Wang Y, Wong E, Sherson SM, et al (2004) P-type ATPase heavy metal transporters with roles in essential zinc homeostasis in Arabidopsis. Plant Cell 16: 1327–1339. 15100400 10.1105/tpc.020487 PMC423219

[9] Valdes B, Duke M, Peaston KA, Lahner B, et al (2010) Functional significance of AtHMA4 C-terminal domain in planta. PLoS ONE 5: e13388 doi:10.1371/journal.pone.0013388. 20975991 10.1371/journal.pone.0013388 PMC2958113

[10] Hanikenne M, Talke IN, Haydon MJ, Lanz C, Nolte A, et al (2008) Evolution of metal hyperaccumulation required cis-regulatory changes and triplication of HMA4. Nature 453: 391–395. 18425111 10.1038/nature06877

[11] Ó Lochlainn S, Bowen HC, Fray RG, Hammond JP, King GJ, et al (2011) Tandem quadruplication of HMA4 in the zinc (Zn) and cadmium (Cd) hyperaccumulator Noccaea caerulescens. PLoS ONE 6: e17814 doi:10.1371/journal.pone.0017814. 21423774 10.1371/journal.pone.0017814 PMC3053397

[12] Satoh-Nagasawa N, Mori M, Nakazawa N, Kawamoto T, Nagato Y, et al (2011) Mutations in rice (Oryza sativa) heavy metal ATPase 2 (OsHMA2) restrict the translocation of Zn and Cd. Plant Cell Physiol 10.1093/pcp/pcr166 22123790

[13] Nocito FF, Lancilli C, Dendena B, Lucchini G, Sacchi GA (2011) Cadmium retention in rice roots is influenced by cadmium availability, chelation and translocation. Plant Cell Environ 34: 994–1008. 21388416 10.1111/j.1365-3040.2011.02299.x

[14] Korenkov V, King B, Hirschi K, Wagner GJ (2009) Root-selective expression of AtCAX4 and AtCAX2 results in reduced lamina cadmium in field-grown Nicotiana tabacum L. Plant Biotechnol J 7: 219–226. 19175521 10.1111/j.1467-7652.2008.00390.x

[15] Koren'kov V, Park S, Cheng NH, Sreevidya C, Lachmansingh J, et al (2007) Enhanced Cd2+ -selective root-tonoplast-transport in tobaccos expressing Arabidopsis cation exchangers. Planta 225: 403–411. 16845524 10.1007/s00425-006-0352-7

[16] Ueno D, Milner MJ, Yamaji N, Yokosho K, Koyama E, et al (2011) Elevated expression of TcHMA3 plays a key role in the extreme Cd tolerance in a Cd-hyperaccumulating ecotype of Thlaspi caerulescens. Plant J 66: 852–862. 21457363 10.1111/j.1365-313X.2011.04548.x

[17] Ueno D, Yamaji N, Kono I, Huang CF, Ando T, et al (2010) Gene limiting cadmium accumulation in rice. Proc Natl Acad Sci U S A 107: 16500–16505. 20823253 10.1073/pnas.1005396107 PMC2944702

[18] Miyadate H, Adachi S, Hiraizumi A, Tezuka K, Nakazawa N, et al (2011) OsHMA3, a P1B-type of ATPase affects root-to-shoot cadmium translocation in rice by mediating efflux into vacuoles. New Phytol 189: 190–199. 20840506 10.1111/j.1469-8137.2010.03459.x

[19] Morel M, Crouzet J, Gravot A, Auroy P, Leonhardt N, et al (2009) AtHMA3, a P1B-ATPase allowing Cd/Zn/Co/Pb vacuolar storage in Arabidopsis. Plant Physiol 149: 894–904. 19036834 10.1104/pp.108.130294 PMC2633814

[20] Park J, Song WY, Ko D, Eom Y, Hansen TH, et al (2012) The phytochelatin transporters AtABCC1 and AtABCC2 mediate tolerance to cadmium and mercury. Plant J 69: 278–288. 21919981 10.1111/j.1365-313X.2011.04789.x

[21] Mendoza-Cozatl DG, Zhai Z, Jobe TO, Akmakjian GZ, Song WY, et al (2011) Tonoplast-localized Abc2 transporter mediates phytochelatin accumulation in vacuoles and confers cadmium tolerance. J Biol Chem 285: 40416–40426. 10.1074/jbc.M110.155408 PMC3003340 20937798

[22] Becher M, Talke IN, Krall L, Kramer U (2004) Cross-species microarray transcript profiling reveals high constitutive expression of metal homeostasis genes in shoots of the zinc hyperaccumulator Arabidopsis halleri. Plant J 37: 251–268. 14690509 10.1046/j.1365-313x.2003.01959.x

[23] Gravot A, Lieutaud A, Verret F, Auroy P, Vavasseur A, et al (2004) AtHMA3, a plant P1B-ATPase, functions as a Cd/Pb transporter in yeast. FEBS Lett 561: 22–28. 15013746 10.1016/S0014-5793(04)00072-9

[24] Alonso-Blanco C, Aarts MG, Bentsink L, Keurentjes JJ, Reymond M, et al (2009) What has natural variation taught us about plant development, physiology, and adaptation? Plant Cell 21: 1877–1896. 19574434 10.1105/tpc.109.068114 PMC2729614

[25] Koornneef M, Alonso-Blanco C, Vreugdenhil D (2004) Naturally occurring genetic variation in Arabidopsis thaliana. Annu Rev Plant Biol 55: 141–172. 15377217 10.1146/annurev.arplant.55.031903.141605

[26] Baxter I, Brazelton JN, Yu D, Huang YS, Lahner B, et al (2010) A coastal cline in sodium accumulation in Arabidopsis thaliana is driven by natural variation of the sodium transporter AtHKT1;1. PLoS Genet 6: e1001193 doi:10.1371/journal.pgen.1001193. 21085628 10.1371/journal.pgen.1001193 PMC2978683

[27] Fournier-Level A, Korte A, Cooper MD, Nordborg M, Schmitt J, et al (2011) A map of local adaptation in Arabidopsis thaliana. Science 334: 86–89. 21980109 10.1126/science.1209271

[28] Hancock AM, Brachi B, Faure N, Horton MW, Jarymowycz LB, et al (2011) Adaptation to climate across the Arabidopsis thaliana genome. Science 334: 83–86. 21980108 10.1126/science.1209244

[29] Horton MW, Hancock AM, Huang YS, Toomajian C, Atwell S, et al (2012) Genome-wide patterns of genetic variation in worldwide Arabidopsis thaliana accessions from the RegMap panel. Nat Genet 2012 44: 212–216. 10.1038/ng.1042 PMC3267885 22231484

[30] Hoffmannm MH (2002) Biogeography of Arabidopsis thaliana (L.) Heynh. (Brassicacceae). J Biogrogr 29: 125–134.

[31] Atwell S, Huang YS, Vilhjalmsson BJ, Willems G, Horton M, et al (2010) Genome-wide association study of 107 phenotypes in Arabidopsis thaliana inbred lines. Nature 465: 627–631. 20336072 10.1038/nature08800 PMC3023908

[32] Li Y, Huang Y, Bergelson J, Nordborg M, Borevitz JO (2010) Association mapping of local climate-sensitive quantitative trait loci in Arabidopsis thaliana. Proc Natl Acad Sci U S A 107: 21199–21204. 21078970 10.1073/pnas.1007431107 PMC3000268

[33] Brachi B, Faure N, Horton M, Flahauw E, Vazquez A, et al (2010) Linkage and association mapping of Arabidopsis thaliana flowering time in nature. PLoS Genet 6: e1000940 doi:10.1371/journal.pgen.1000940. 20463887 10.1371/journal.pgen.1000940 PMC2865524

[34] Filiault D, Maloof J (2012) A Genome-Wide Association Study Identifies Variants Underlying the *Arabidopsis thaliana* Shade Avoidance Response. PLoS Genet 8: e1002589 doi:10.1371/journal.pgen.1002589. 22438834 10.1371/journal.pgen.1002589 PMC3305432

[35] Aranzana MJ, Kim S, Zhao K, Bakker E, Horton M, et al (2005) Genome-wide association mapping in Arabidopsis identifies previously known flowering time and pathogen resistance genes. PLoS Genet 1: e60 doi:10.1371/journal.pgen.0010060. 16292355 10.1371/journal.pgen.0010060 PMC1283159

[36] Nemri A, Atwell S, Tarone AM, Huang YS, Zhao K, et al (2010) Genome-wide survey of Arabidopsis natural variation in downy mildew resistance using combined association and linkage mapping. Proc Natl Acad Sci U S A 107: 10302–10307. 20479233 10.1073/pnas.0913160107 PMC2890483

[37] Todesco M, Balasubramanian S, Hu TT, Traw MB, Horton M, et al (2010) Natural allelic variation underlying a major fitness trade-off in Arabidopsis thaliana. Nature 465: 632–636. 20520716 10.1038/nature09083 PMC3055268

[38] Huang X, Wei X, Sang T, Zhao Q, Feng Q, et al (2010) Genome-wide association studies of 14 agronomic traits in rice landraces. Nat Genet 42: 961–967. 20972439 10.1038/ng.695

[39] Zhao K, Tung CW, Eizenga GC, Wright MH, Ali ML, et al (2011) Genome-wide association mapping reveals a rich genetic architecture of complex traits in Oryza sativa. Nat Commun 2: 467. 21915109 10.1038/ncomms1467 PMC3195253

[40] Huang X, Zhao Y, Wei X, Li C, Wang A, et al (2011) Genome-wide association study of flowering time and grain yield traits in a worldwide collection of rice germplasm. Nat Genet 44: 32–39. 22138690 10.1038/ng.1018

[41] Kump KL, Bradbury PJ, Wisser RJ, Buckler ES, Belcher AR, et al (2011) Genome-wide association study of quantitative resistance to southern leaf blight in the maize nested association mapping population. Nat Genet 43: 163–168. 21217757 10.1038/ng.747

[42] Tian F, Bradbury PJ, Brown PJ, Hung H, Sun Q, et al (2011) Genome-wide association study of leaf architecture in the maize nestedassociation mapping population. Nat Genet 43: 159–162. 21217756 10.1038/ng.746

[43] Lahner B, Gong J, Mahmoudian M, Smith EL, Abid KB, et al (2003) Genomic scale profiling of nutrient and trace elements in Arabidopsis thaliana. Nat Biotechnol 21: 1215–1221. 12949535 10.1038/nbt865

[44] Yu J, Pressoir G, Briggs WH, Vroh Bi I, Yamasaki M, et al (2006) A unified mixed-model method for association mapping that accounts for multiple levels of relatedness. Nat Genet 38: 203–208. 16380716 10.1038/ng1702

[45] Beck JB, Schmuths H, Schaal BA (2008) Native range genetic variation in Arabidopsis thaliana is strongly geographically structured and reflects Pleistocene glacial dynamics. Mol Ecol 17: 902–915. 18179426 10.1111/j.1365-294X.2007.03615.x

[46] Wolyn DJ, Borevitz JO, Loudet O, Schwartz C, Maloof J, et al (2004) Light-response quantitative trait loci identified with composite interval and eXtreme array mapping in Arabidopsis thaliana. Genetics 167: 907–917. 15238539 10.1534/genetics.103.024810 PMC1470895

[47] Becker A, Chao DY, Zhang X, Salt DE, Baxter I (2011) Bulk segregant analysis using single nucleotide polymorphism microarrays. PLoS ONE 6: e15993 doi:10.1371/journal.pone.0015993. 21297997 10.1371/journal.pone.0015993 PMC3029305

[48] Platt A, Horton M, Huang YS, Li Y, Anastasio AE, et al (2010) The scale of population structure in Arabidopsis thaliana. PLoS Genet 6: e1000843 doi:10.1371/journal.pgen.1000843. 20169178 10.1371/journal.pgen.1000843 PMC2820523

[49] Rus A, Baxter I, Muthukumar B, Gustin J, Lahner B, et al (2006) Natural variants of AtHKT1 enhance Na+ accumulation in two wild populations of Arabidopsis. PLoS Genet 2: e210 doi:10.1371/journal.pgen.0020210. 17140289 10.1371/journal.pgen.0020210 PMC1665649

[50] Baxter I, Muthukumar B, Park HC, Buchner P, Lahner B, et al (2008) Variation in molybdenum content across broadly distributed populations of Arabidopsis thaliana is controlled by a mitochondrial molybdenum transporter (MOT1). PLoS Genet 4: e1000004 doi:10.1371/journal.pgen.1000004. 18454190 10.1371/journal.pgen.1000004 PMC2265440

[51] Baxter I, Ouzzani M, Orcun S, Kennedy B, Jandhyala SS, et al (2007) Purdue ionomics information management system. An integrated functional genomics platform. Plant Physiol 143: 600–611. 17189337 10.1104/pp.106.092528 PMC1803751

[52] Borevitz JO, Liang D, Plouffe D, Chang HS, Zhu T, et al (2003) Large-scale identification of single-feature polymorphisms in complex genomes. Genome Res 13: 513–523. 12618383 10.1101/gr.541303 PMC430246

[53] Clough SJ, Bent AF (1998) Floral dip: a simplified method for Agrobacterium-mediated transformation of Arabidopsis thaliana. Plant J 16: 735–743. 10069079 10.1046/j.1365-313x.1998.00343.x

[54] Livak KJ, Schmittgen TD (2001) Analysis of relative gene expression data using real-time quantitative PCR and the 2(-Delta Delta C(T)). Method Methods 25: 402–408. 11846609 10.1006/meth.2001.1262

