# Supplementary figures and images for: Genome-Wide Association Studies Identify Heavy Metal ATPase3 as the Primary Determinant of Natural Variation in Leaf Cadmium in Arabidopsis thaliana
Source: PLoS Genet. 2012 Sep 6;8(9):e1002923. doi: 10.1371/journal.pgen.1002923 (PMC3435251; doi:10.1371/journal.pgen.1002923)

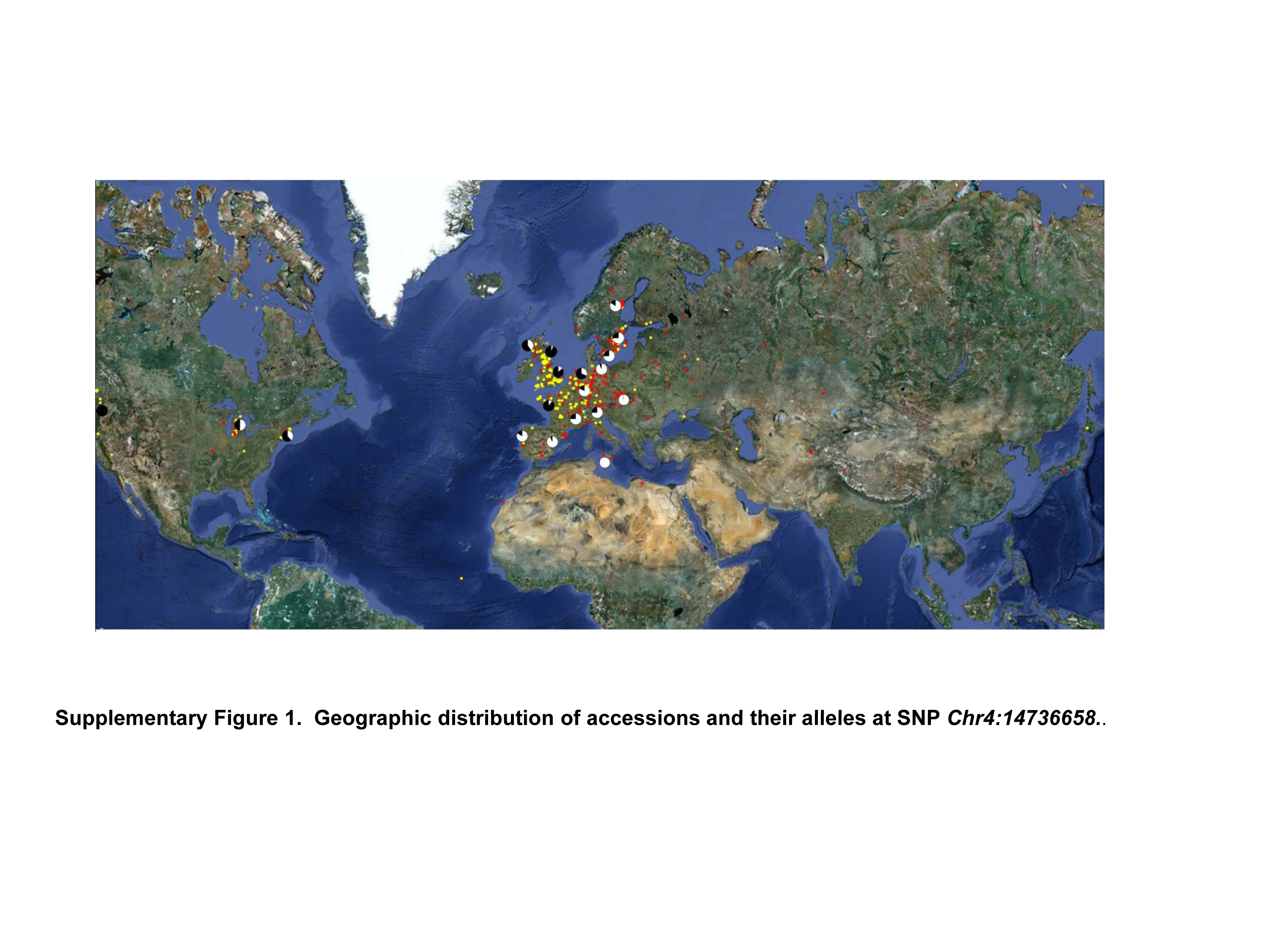

Supplement: Figure S1 — Geographic distribution of accessions and their alleles at the SNP Chr4:14736658. Map showing the geographical position of the collection site of 1178 accessions of A. thaliana. The genotype at Chr4:14736658 of each accession is represented by the type of symbol (red Chr4:6392276 = C, yellow Chr4:6392276 = T). Pie charts on the map represent the proportion of the local population containing the T allele (black sector) and C allele (white sector).Total 19 local populations (West USA, Middle-east USA, East USA, North UK, Middle UK, South UK, West France, East France, Portugal, Spain, Netherland, Middle Germany, North Germany, South Sweden, Middle Sweden, North Sweden, Czech Republic, The alps and South Italy) are plotted. (TIF) [file pgen.1002923.s001.tif]

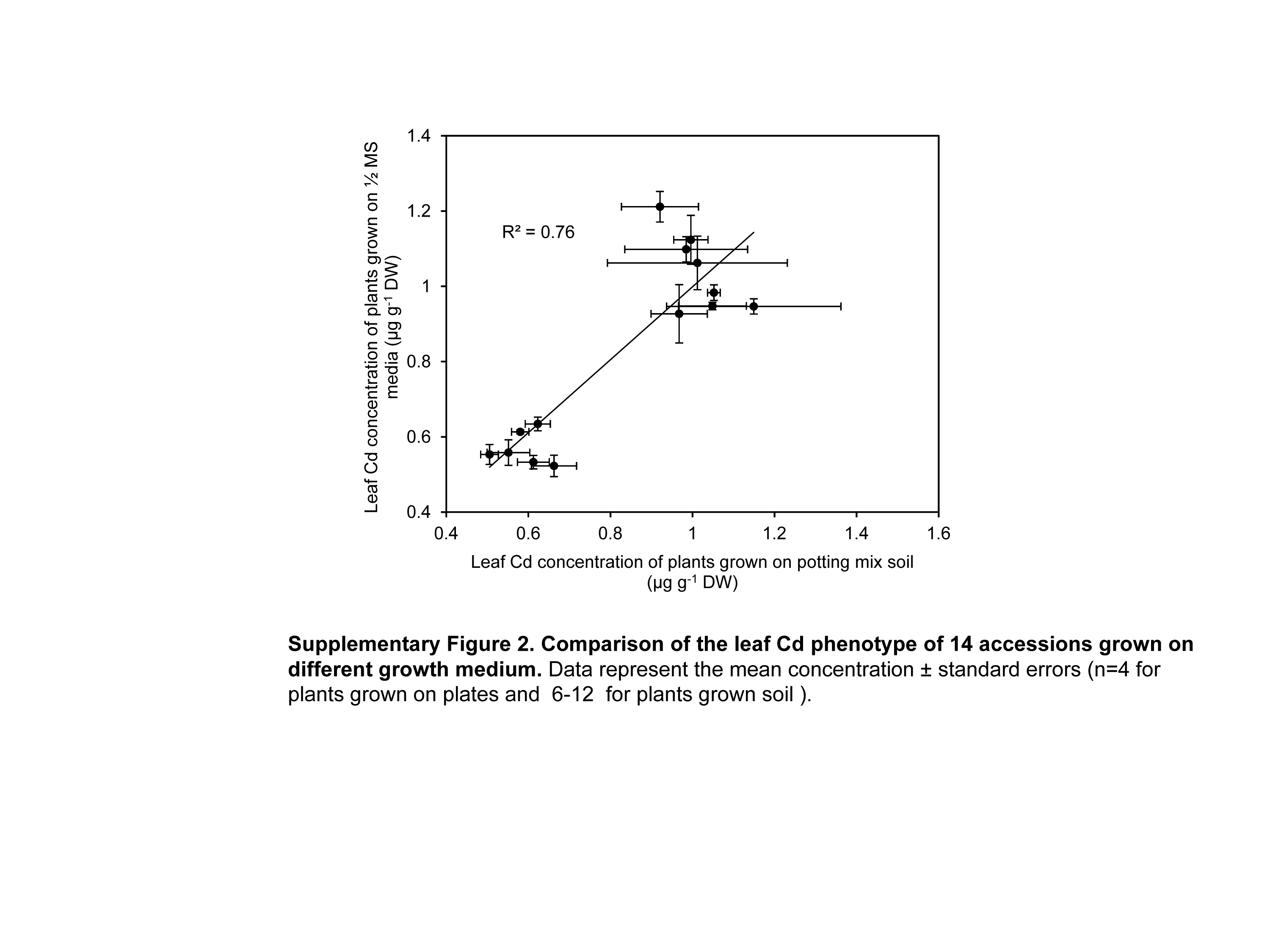

Supplement: Figure S2 — Comparison of the leaf Cd concentration in 14 A. thaliana accessions grown on different growth medium. Data represent the mean leaf Cd concentration ± standard errors (n = 4 for plants grown on solidified ½ MS media and 6–12 for plants grown on potting mix soil). (TIF) [file pgen.1002923.s002.tif]
